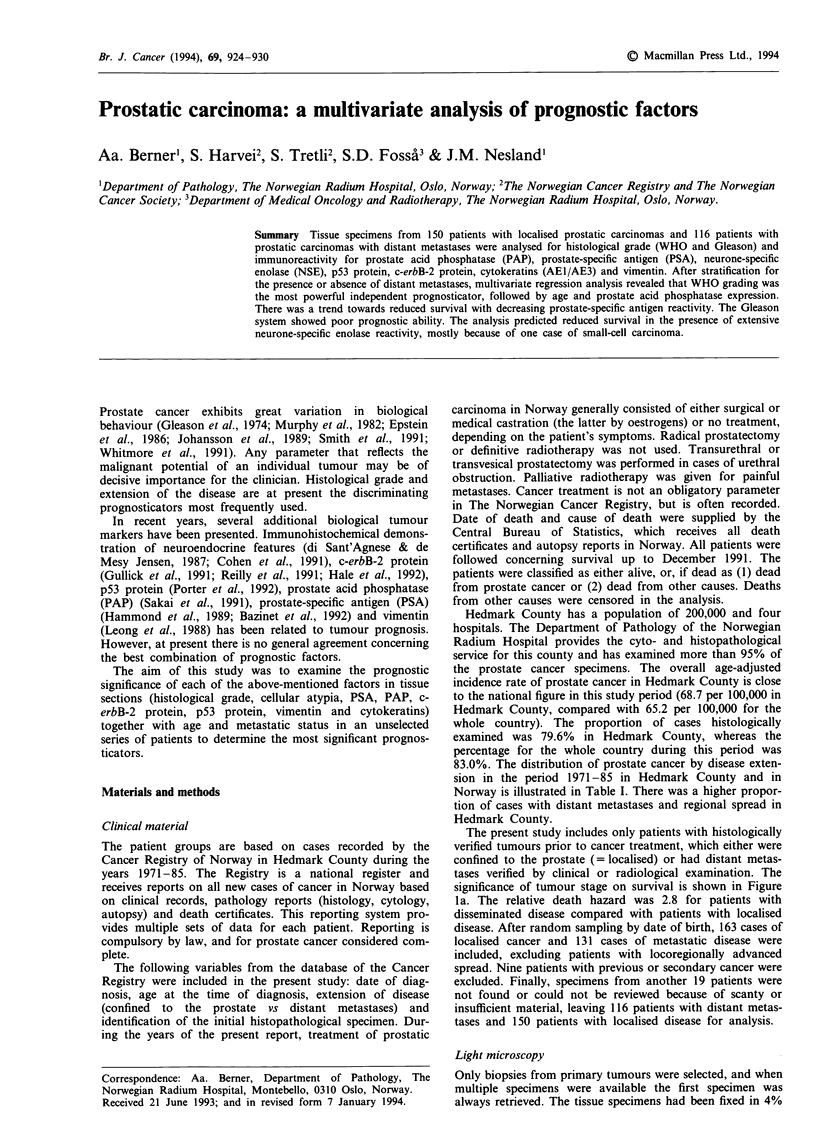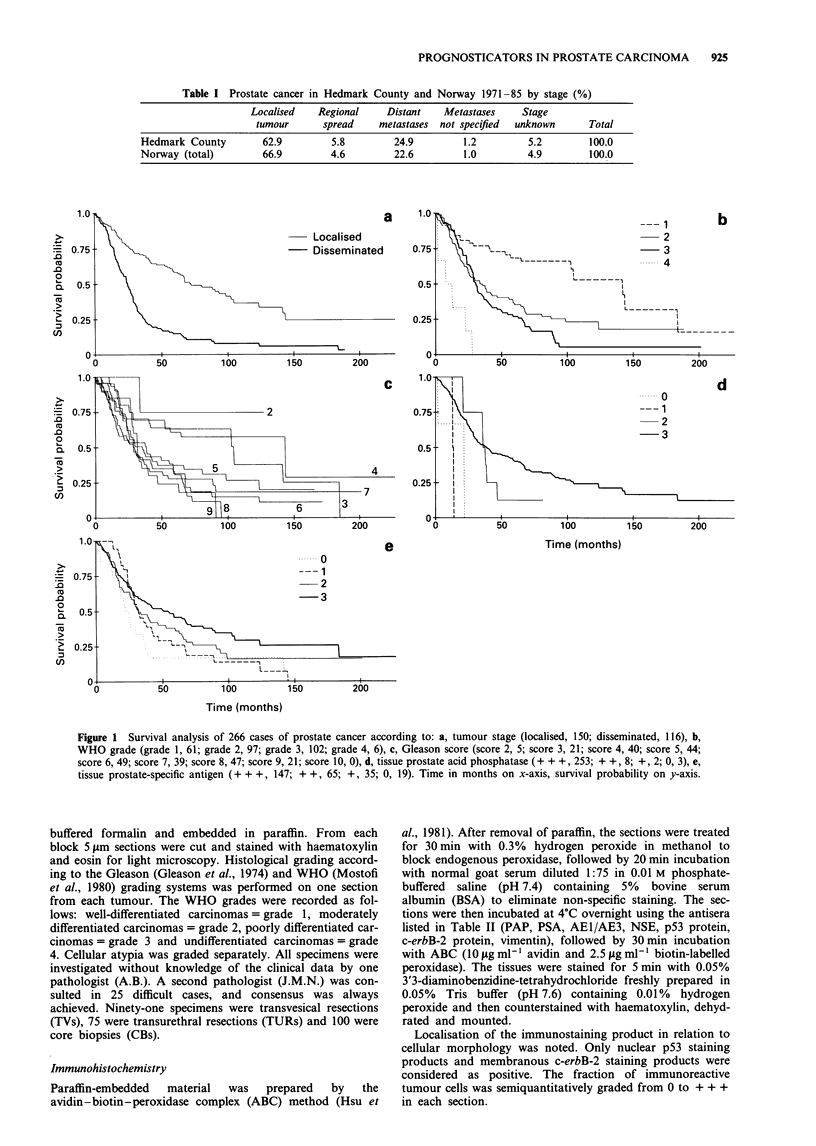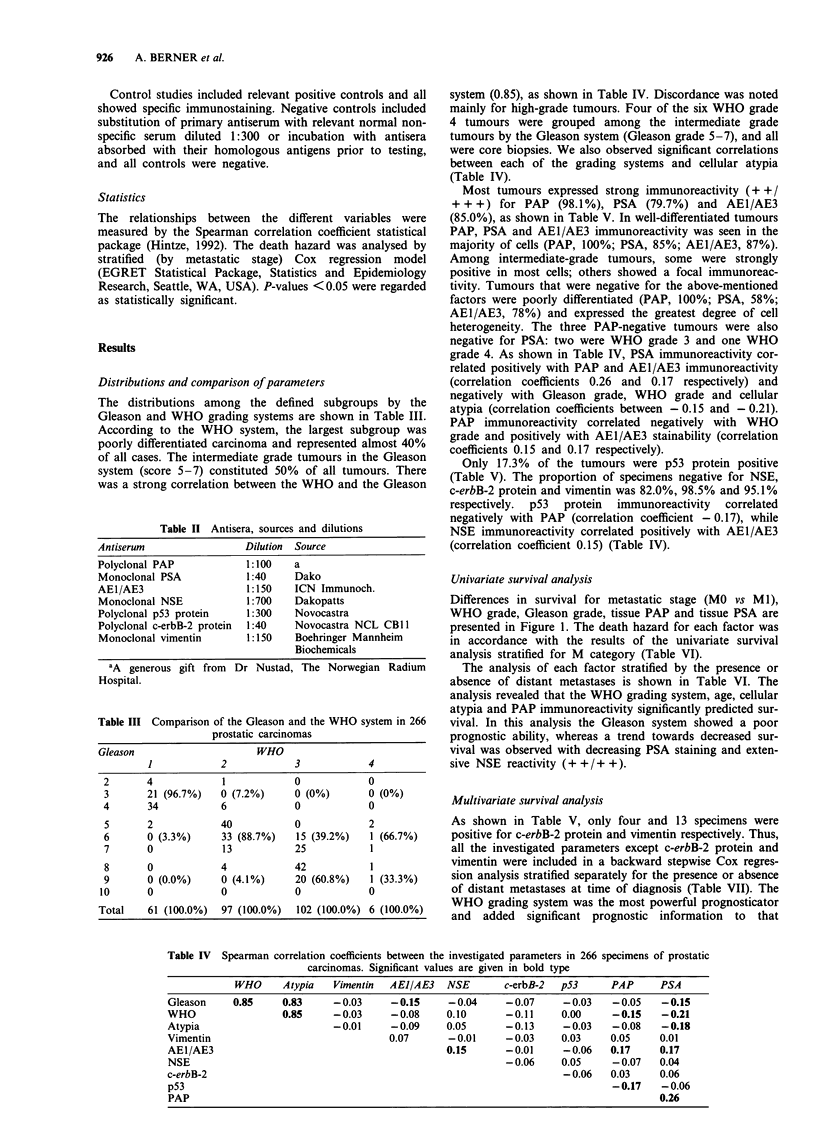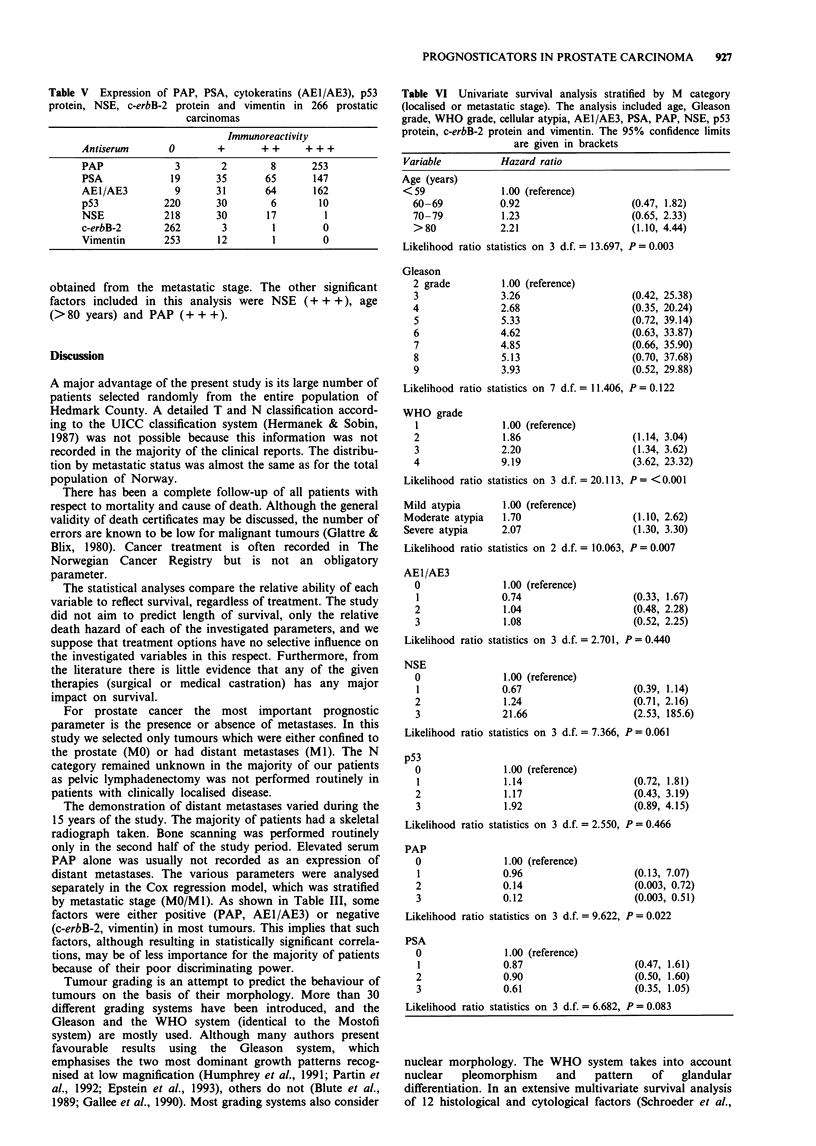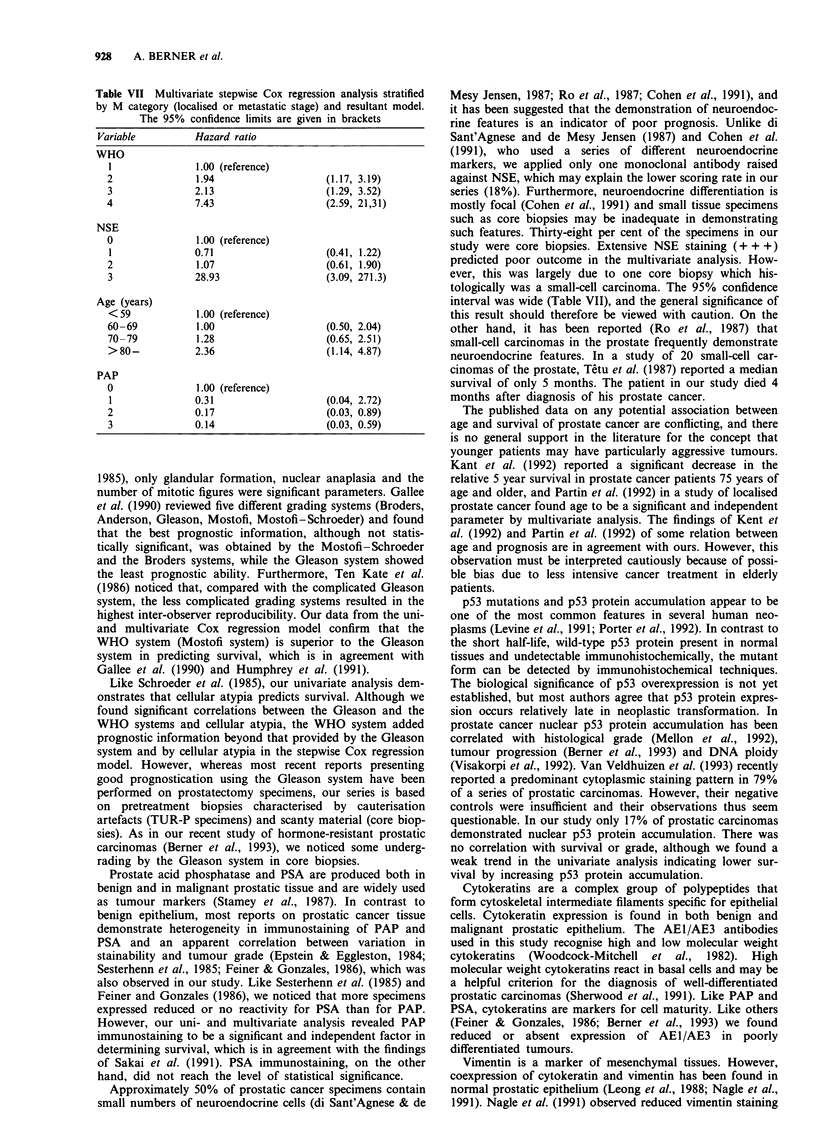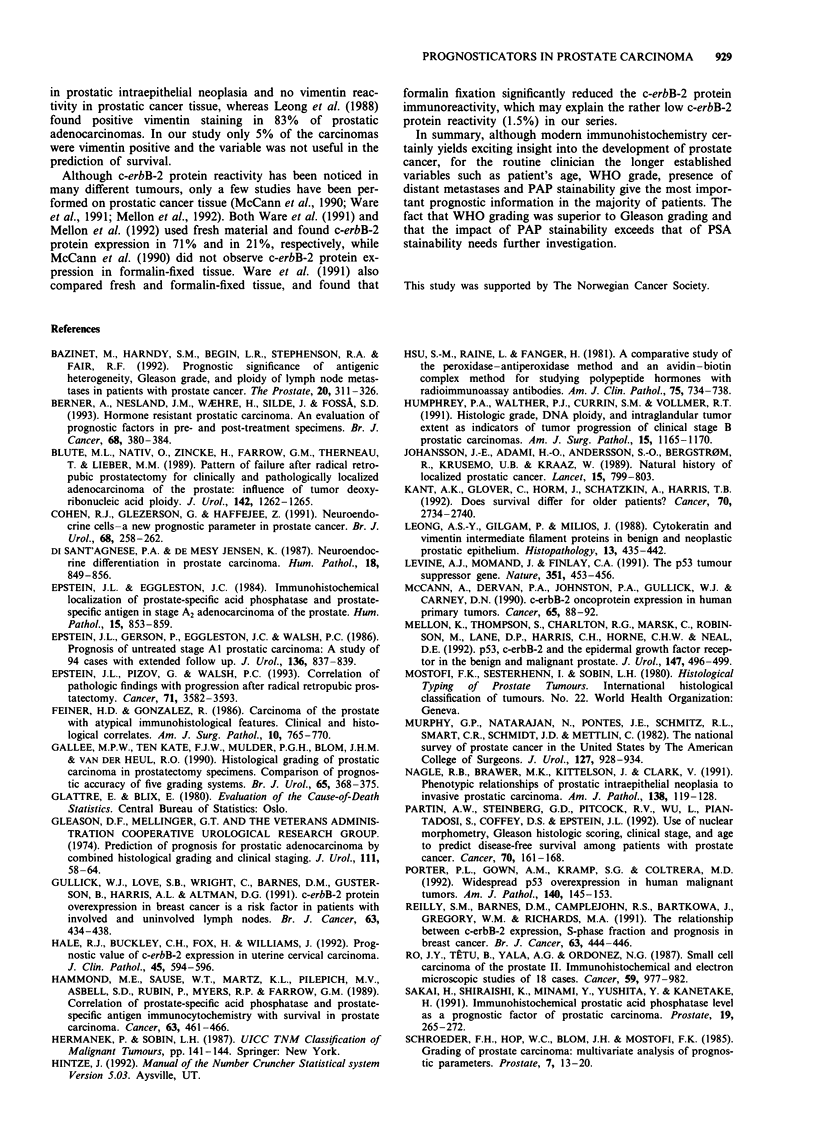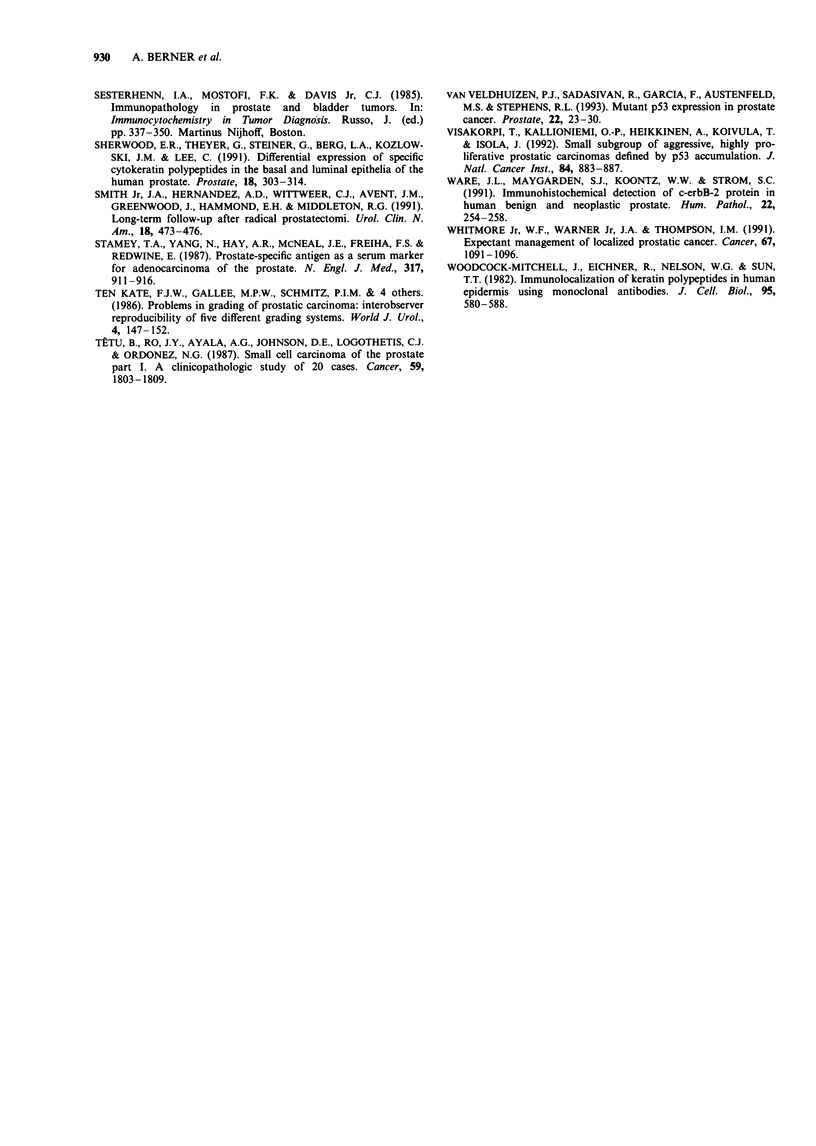# Prostatic carcinoma: a multivariate analysis of prognostic factors.

**DOI:** 10.1038/bjc.1994.179

**Published:** 1994-05

**Authors:** A. Berner, S. Harvei, S. Tretli, S. D. Fosså, J. M. Nesland

**Affiliations:** Department of Pathology, Norwegian Radium Hospital, Oslo.

## Abstract

Tissue specimens from 150 patients with localised prostatic carcinomas and 116 patients with prostatic carcinomas with distant metastases were analysed for histological grade (WHO and Gleason) and immunoreactivity for prostate acid phosphatase (PAP), prostate-specific antigen (PSA), neurone-specific enolase (NSE), p53 protein, c-erbB-2 protein, cytokeratins (AE1/AE3) and vimentin. After stratification for the presence or absence of distant metastases, multivariate regression analysis revealed that WHO grading was the most powerful independent prognosticator, followed by age and prostate acid phosphatase expression. There was a trend towards reduced survival with decreasing prostate-specific antigen reactivity. The Gleason system showed poor prognostic ability. The analysis predicted reduced survival in the presence of extensive neurone-specific enolase reactivity, mostly because of one case of small-cell carcinoma.


*Br. J. Cancer* (1994), **69**, 924–930 © Macmillan Press Ltd., 1994

### Prostatic carcinoma: a multivariate analysis of prognostic factors

Aa. Berner[1], S. Harvei[2], S. Tretli[2], S.D. Fosså[3] & J.M. Nesland[1]

[1]*Department of Pathology, The Norwegian Radium Hospital, Oslo, Norway;* [2]*The Norwegian Cancer Registry and The Norwegian Cancer Society;* [3]*Department of Medical Oncology and Radiotherapy, The Norwegian Radium Hospital, Oslo, Norway.*

**Summary** Tissue specimens from 150 patients with localised prostatic carcinomas and 116 patients with prostatic carcinomas with distant metastases were analysed for histological grade (WHO and Gleason) and immunoreactivity for prostate acid phosphatase (PAP), prostate-specific antigen (PSA), neurone-specific enolase (NSE), p53 protein, c-*erb*B-2 protein, cytokeratins (AE1/AE3) and vimentin. After stratification for the presence or absence of distant metastases, multivariate regression analysis revealed that WHO grading was the most powerful independent prognosticator, followed by age and prostate acid phosphatase expression. There was a trend towards reduced survival with decreasing prostate-specific antigen reactivity. The Gleason system showed poor prognostic ability. The analysis predicted reduced survival in the presence of extensive neurone-specific enolase reactivity, mostly because of one case of small-cell carcinoma.

Prostate cancer exhibits great variation in biological behaviour (Gleason *et al.*, 1974; Murphy *et al.*, 1982; Epstein *et al.*, 1986; Johansson *et al.*, 1989; Smith *et al.*, 1991; Whitmore *et al.*, 1991). Any parameter that reflects the malignant potential of an individual tumour may be of decisive importance for the clinician. Histological grade and extension of the disease are at present the discriminating prognosticators most frequently used.

In recent years, several additional biological tumour markers have been presented. Immunohistochemical demonstration of neuroendocrine features (di Sant'Agnese & de Mesy Jensen, 1987; Cohen *et al.*, 1991), c-*erb*B-2 protein (Gullick *et al.*, 1991; Reilly *et al.*, 1991; Hale *et al.*, 1992), p53 protein (Porter *et al.*, 1992), prostate acid phosphatase (PAP) (Sakai *et al.*, 1991), prostate-specific antigen (PSA) (Hammond *et al.*, 1989; Bazinet *et al.*, 1992) and vimentin (Leong *et al.*, 1988) has been related to tumour prognosis. However, at present there is no general agreement concerning the best combination of prognostic factors.

The aim of this study was to examine the prognostic significance of each of the above-mentioned factors in tissue sections (histological grade, cellular atypia, PSA, PAP, c-*erb*B-2 protein, p53 protein, vimentin and cytokeratins) together with age and metastatic status in an unselected series of patients to determine the most significant prognosticators.

### Materials and methods

### *Clinical material*

The patient groups are based on cases recorded by the Cancer Registry of Norway in Hedmark County during the years 1971–85. The Registry is a national register and receives reports on all new cases of cancer in Norway based on clinical records, pathology reports (histology, cytology, autopsy) and death certificates. This reporting system provides multiple sets of data for each patient. Reporting is compulsory by law, and for prostate cancer considered complete.

The following variables from the database of the Cancer Registry were included in the present study: date of diagnosis, age at the time of diagnosis, extension of disease (confined to the prostate *vs* distant metastases) and identification of the initial histopathological specimen. During the years of the present report, treatment of prostatic carcinoma in Norway generally consisted of either surgical or medical castration (the latter by oestrogens) or no treatment, depending on the patient's symptoms. Radical prostatectomy or definitive radiotherapy was not used. Transurethral or transvesical prostatectomy was performed in cases of urethral obstruction. Palliative radiotherapy was given for painful metastases. Cancer treatment is not an obligatory parameter in The Norwegian Cancer Registry, but is often recorded. Date of death and cause of death were supplied by the Central Bureau of Statistics, which receives all death certificates and autopsy reports in Norway. All patients were followed concerning survival up to December 1991. The patients were classified as either alive, or, if dead as (1) dead from prostate cancer or (2) dead from other causes. Deaths from other causes were censored in the analysis.

Hedmark County has a population of 200,000 and four hospitals. The Department of Pathology of the Norwegian Radium Hospital provides the cyto- and histopathological service for this county and has examined more than 95% of the prostate cancer specimens. The overall age-adjusted incidence rate of prostate cancer in Hedmark County is close to the national figure in this study period (68.7 per 100,000 in Hedmark County, compared with 65.2 per 100,000 for the whole country). The proportion of cases histologically examined was 79.6% in Hedmark County, whereas the percentage for the whole country during this period was 83.0%. The distribution of prostate cancer by disease extension in the period 1971–85 in Hedmark County and in Norway is illustrated in Table I. There was a higher proportion of cases with distant metastases and regional spread in Hedmark County.

The present study includes only patients with histologically verified tumours prior to cancer treatment, which either were confined to the prostate (= localised) or had distant metastases verified by clinical or radiological examination. The significance of tumour stage on survival is shown in Figure 1a. The relative death hazard was 2.8 for patients with disseminated disease compared with patients with localised disease. After random sampling by date of birth, 163 cases of localised cancer and 131 cases of metastatic disease were included, excluding patients with locoregionally advanced spread. Nine patients with previous or secondary cancer were excluded. Finally, specimens from another 19 patients were not found or could not be reviewed because of scanty or insufficient material, leaving 116 patients with distant metastases and 150 patients with localised disease for analysis.

### *Light microscopy*

Only biopsies from primary tumours were selected, and when multiple specimens were available the first specimen was always retrieved. The tissue specimens had been fixed in 4%

Correspondence: Aa. Berner, Department of Pathology, The Norwegian Radium Hospital, Montebello, 0310 Oslo, Norway.
Received 21 June 1993; and in revised form 7 January 1994.

**Table I** Prostate cancer in Hedmark County and Norway 1971–85 by stage (%)

| | *Localised tumour* | *Regional spread* | *Distant metastases* | *Metastases not specified* | *Stage unknown* | *Total* |
|---|---|---|---|---|---|---|
| Hedmark County | 62.9 | 5.8 | 24.9 | 1.2 | 5.2 | 100.0 |
| Norway (total) | 66.9 | 4.6 | 22.6 | 1.0 | 4.9 | 100.0 |

**Figure 1** Survival analysis of 266 cases of prostate cancer according to: **a**, tumour stage (localised, 150; disseminated, 116), **b**, WHO grade (grade 1, 61; grade 2, 97; grade 3, 102; grade 4, 6), **c**, Gleason score (score 2, 5; score 3, 21; score 4, 40; score 5, 44; score 6, 49; score 7, 39; score 8, 47; score 9, 21; score 10, 0), **d**, tissue prostate acid phosphatase (+ + +, 253; + +, 8; +, 2; 0, 3), **e**, tissue prostate-specific antigen (+ + +, 147; + +, 65; +, 35; 0, 19). Time in months on *x*-axis, survival probability on *y*-axis.

buffered formalin and embedded in paraffin. From each block 5 µm sections were cut and stained with haematoxylin and eosin for light microscopy. Histological grading according to the Gleason (Gleason *et al.*, 1974) and WHO (Mostofi *et al.*, 1980) grading systems was performed on one section from each tumour. The WHO grades were recorded as follows: well-differentiated carcinomas = grade 1, moderately differentiated carcinomas = grade 2, poorly differentiated carcinomas = grade 3 and undifferentiated carcinomas = grade 4. Cellular atypia was graded separately. All specimens were investigated without knowledge of the clinical data by one pathologist (A.B.). A second pathologist (J.M.N.) was consulted in 25 difficult cases, and consensus was always achieved. Ninety-one specimens were transvesical resections (TVs), 75 were transurethral resections (TURs) and 100 were core biopsies (CBs).

*Immunohistochemistry*

Paraffin-embedded material was prepared by the avidin–biotin–peroxidase complex (ABC) method (Hsu *et al.*, 1981). After removal of paraffin, the sections were treated for 30 min with 0.3% hydrogen peroxide in methanol to block endogenous peroxidase, followed by 20 min incubation with normal goat serum diluted 1:75 in 0.01 M phosphate-buffered saline (pH 7.4) containing 5% bovine serum albumin (BSA) to eliminate non-specific staining. The sections were then incubated at 4°C overnight using the antisera listed in Table II (PAP, PSA, AE1/AE3, NSE, p53 protein, c-*erb*B-2 protein, vimentin), followed by 30 min incubation with ABC (10 µg ml$^{-1}$ avidin and 2.5 µg ml$^{-1}$ biotin-labelled peroxidase). The tissues were stained for 5 min with 0.05% 3′3-diaminobenzidine-tetrahydrochloride freshly prepared in 0.05% Tris buffer (pH 7.6) containing 0.01% hydrogen peroxide and then counterstained with haematoxylin, dehydrated and mounted.

Localisation of the immunostaining product in relation to cellular morphology was noted. Only nuclear p53 staining products and membranous c-*erb*B-2 staining products were considered as positive. The fraction of immunoreactive tumour cells was semiquantitatively graded from 0 to + + + in each section.

Control studies included relevant positive controls and all showed specific immunostaining. Negative controls included substitution of primary antiserum with relevant normal non-specific serum diluted 1:300 or incubation with antisera absorbed with their homologous antigens prior to testing, and all controls were negative.

*Statistics*

The relationships between the different variables were measured by the Spearman correlation coefficient statistical package (Hintze, 1992). The death hazard was analysed by stratified (by metastatic stage) Cox regression model (EGRET Statistical Package, Statistics and Epidemiology Research, Seattle, WA, USA). *P*-values <0.05 were regarded as statistically significant.

### Results

*Distributions and comparison of parameters*

The distributions among the defined subgroups by the Gleason and WHO grading systems are shown in Table III. According to the WHO system, the largest subgroup was poorly differentiated carcinoma and represented almost 40% of all cases. The intermediate grade tumours in the Gleason system (score 5–7) constituted 50% of all tumours. There was a strong correlation between the WHO and the Gleason system (0.85), as shown in Table IV. Discordance was noted mainly for high-grade tumours. Four of the six WHO grade 4 tumours were grouped among the intermediate grade tumours by the Gleason system (Gleason grade 5–7), and all were core biopsies. We also observed significant correlations between each of the grading systems and cellular atypia (Table IV).

Most tumours expressed strong immunoreactivity (++/+++) for PAP (98.1%), PSA (79.7%) and AE1/AE3 (85.0%), as shown in Table V. In well-differentiated tumours PAP, PSA and AE1/AE3 immunoreactivity was seen in the majority of cells (PAP, 100%; PSA, 85%; AE1/AE3, 87%). Among intermediate-grade tumours, some were strongly positive in most cells; others showed a focal immunoreactivity. Tumours that were negative for the above-mentioned factors were poorly differentiated (PAP, 100%; PSA, 58%; AE1/AE3, 78%) and expressed the greatest degree of cell heterogeneity. The three PAP-negative tumours were also negative for PSA: two were WHO grade 3 and one WHO grade 4. As shown in Table IV, PSA immunoreactivity correlated positively with PAP and AE1/AE3 immunoreactivity (correlation coefficients 0.26 and 0.17 respectively) and negatively with Gleason grade, WHO grade and cellular atypia (correlation coefficients between −0.15 and −0.21). PAP immunoreactivity correlated negatively with WHO grade and positively with AE1/AE3 stainability (correlation coefficients 0.15 and 0.17 respectively).

Only 17.3% of the tumours were p53 protein positive (Table V). The proportion of specimens negative for NSE, c-*erb*B-2 protein and vimentin was 82.0%, 98.5% and 95.1% respectively. p53 protein immunoreactivity correlated negatively with PAP (correlation coefficient −0.17), while NSE immunoreactivity correlated positively with AE1/AE3 (correlation coefficient 0.15) (Table IV).

*Univariate survival analysis*

Differences in survival for metastatic stage (M0 *vs* M1), WHO grade, Gleason grade, tissue PAP and tissue PSA are presented in Figure 1. The death hazard for each factor was in accordance with the results of the univariate survival analysis stratified for M category (Table VI).

The analysis of each factor stratified by the presence or absence of distant metastases is shown in Table VI. The analysis revealed that the WHO grading system, age, cellular atypia and PAP immunoreactivity significantly predicted survival. In this analysis the Gleason system showed a poor prognostic ability, whereas a trend towards decreased survival was observed with decreasing PSA staining and extensive NSE reactivity (++/++).

*Multivariate survival analysis*

As shown in Table V, only four and 13 specimens were positive for c-*erb*B-2 protein and vimentin respectively. Thus, all the investigated parameters except c-*erb*B-2 protein and vimentin were included in a backward stepwise Cox regression analysis stratified separately for the presence or absence of distant metastases at time of diagnosis (Table VII). The WHO grading system was the most powerful prognosticator and added significant prognostic information to that

**Table II** Antisera, sources and dilutions

| *Antiserum* | *Dilution* | *Source* |
|---|---|---|
| Polyclonal PAP | 1:100 | a |
| Monoclonal PSA | 1:40 | Dako |
| AE1/AE3 | 1:150 | ICN Immunoch. |
| Monoclonal NSE | 1:700 | Dakopatts |
| Polyclonal p53 protein | 1:300 | Novocastra |
| Polyclonal c-erbB-2 protein | 1:40 | Novocastra NCL CB11 |
| Monoclonal vimentin | 1:150 | Boehringer Mannheim Biochemicals |

[a]A generous gift from Dr Nustad, The Norwegian Radium Hospital.

**Table III** Comparison of the Gleason and the WHO system in 266 prostatic carcinomas

| *Gleason* | *WHO* *1* | *2* | *3* | *4* |
|---|---|---|---|---|
| 2 | 4 | 1 | 0 | 0 |
| 3 | 21 (96.7%) | 0 (7.2%) | 0 (0%) | 0 (0%) |
| 4 | 34 | 6 | 0 | 0 |
| 5 | 2 | 40 | 0 | 2 |
| 6 | 0 (3.3%) | 33 (88.7%) | 15 (39.2%) | 1 (66.7%) |
| 7 | 0 | 13 | 25 | 1 |
| 8 | 0 | 4 | 42 | 1 |
| 9 | 0 (0.0%) | 0 (4.1%) | 20 (60.8%) | 1 (33.3%) |
| 10 | 0 | 0 | 0 | 0 |
| Total | 61 (100.0%) | 97 (100.0%) | 102 (100.0%) | 6 (100.0%) |

**Table IV** Spearman correlation coefficients between the investigated parameters in 266 specimens of prostatic carcinomas. Significant values are given in bold type

| | *WHO* | *Atypia* | *Vimentin* | *AE1/AE3* | *NSE* | *c-*erb*B-2* | *p53* | *PAP* | *PSA* |
|---|---|---|---|---|---|---|---|---|---|
| Gleason | **0.85** | **0.83** | −0.03 | **−0.15** | −0.04 | −0.07 | −0.03 | −0.05 | **−0.15** |
| WHO | | **0.85** | −0.03 | −0.08 | 0.10 | −0.11 | 0.00 | **−0.15** | **−0.21** |
| Atypia | | | −0.01 | −0.09 | 0.05 | −0.13 | −0.03 | −0.08 | **−0.18** |
| Vimentin | | | | 0.07 | −0.01 | −0.03 | 0.03 | 0.05 | 0.01 |
| AE1/AE3 | | | | | **0.15** | −0.01 | −0.06 | **0.17** | **0.17** |
| NSE | | | | | | −0.06 | 0.05 | −0.07 | 0.04 |
| c-*erb*B-2 | | | | | | | −0.06 | 0.03 | 0.06 |
| p53 | | | | | | | | **−0.17** | −0.06 |
| PAP | | | | | | | | | **0.26** |

**Table V** Expression of PAP, PSA, cytokeratins (AE1/AE3), p53 protein, NSE, c-*erb*B-2 protein and vimentin in 266 prostatic carcinomas

| | Immunoreactivity | | | |
|---|---|---|---|---|
| *Antiserum* | *0* | *+* | *++* | *+++* |
| PAP | 3 | 2 | 8 | 253 |
| PSA | 19 | 35 | 65 | 147 |
| AE1/AE3 | 9 | 31 | 64 | 162 |
| p53 | 220 | 30 | 6 | 10 |
| NSE | 218 | 30 | 17 | 1 |
| c-*erb*B-2 | 262 | 3 | 1 | 0 |
| Vimentin | 253 | 12 | 1 | 0 |

obtained from the metastatic stage. The other significant factors included in this analysis were NSE (+++), age (>80 years) and PAP (+++).

### Discussion

A major advantage of the present study is its large number of patients selected randomly from the entire population of Hedmark County. A detailed T and N classification according to the UICC classification system (Hermanek & Sobin, 1987) was not possible because this information was not recorded in the majority of the clinical reports. The distribution by metastatic status was almost the same as for the total population of Norway.

There has been a complete follow-up of all patients with respect to mortality and cause of death. Although the general validity of death certificates may be discussed, the number of errors are known to be low for malignant tumours (Glattre & Blix, 1980). Cancer treatment is often recorded in The Norwegian Cancer Registry but is not an obligatory parameter.

The statistical analyses compare the relative ability of each variable to reflect survival, regardless of treatment. The study did not aim to predict length of survival, only the relative death hazard of each of the investigated parameters, and we suppose that treatment options have no selective influence on the investigated variables in this respect. Furthermore, from the literature there is little evidence that any of the given therapies (surgical or medical castration) has any major impact on survival.

For prostate cancer the most important prognostic parameter is the presence or absence of metastases. In this study we selected only tumours which were either confined to the prostate (M0) or had distant metastases (M1). The N category remained unknown in the majority of our patients as pelvic lymphadenectomy was not performed routinely in patients with clinically localised disease.

The demonstration of distant metastases varied during the 15 years of the study. The majority of patients had a skeletal radiograph taken. Bone scanning was performed routinely only in the second half of the study period. Elevated serum PAP alone was usually not recorded as an expression of distant metastases. The various parameters were analysed separately in the Cox regression model, which was stratified by metastatic stage (M0/M1). As shown in Table III, some factors were either positive (PAP, AE1/AE3) or negative (c-*erb*B-2, vimentin) in most tumours. This implies that such factors, although resulting in statistically significant correlations, may be of less importance for the majority of patients because of their poor discriminating power.

Tumour grading is an attempt to predict the behaviour of tumours on the basis of their morphology. More than 30 different grading systems have been introduced, and the Gleason and the WHO system (identical to the Mostofi system) are mostly used. Although many authors present favourable results using the Gleason system, which emphasises the two most dominant growth patterns recognised at low magnification (Humphrey *et al.*, 1991; Partin *et al.*, 1992; Epstein *et al.*, 1993), others do not (Blute *et al.*, 1989; Gallee *et al.*, 1990). Most grading systems also consider nuclear morphology. The WHO system takes into account nuclear pleomorphism and pattern of glandular differentiation. In an extensive multivariate survival analysis of 12 histological and cytological factors (Schroeder *et al.*,

**Table VI** Univariate survival analysis stratified by M category (localised or metastatic stage). The analysis included age, Gleason grade, WHO grade, cellular atypia, AE1/AE3, PSA, PAP, NSE, p53 protein, c-*erb*B-2 protein and vimentin. The 95% confidence limits are given in brackets

| *Variable* | *Hazard ratio* | |
|---|---|---|
| Age (years) | | |
| <59 | 1.00 (reference) | |
| 60–69 | 0.92 | (0.47, 1.82) |
| 70–79 | 1.23 | (0.65, 2.33) |
| >80 | 2.21 | (1.10, 4.44) |
| Likelihood ratio statistics on 3 d.f. = 13.697, $P = 0.003$ | | |
| Gleason | | |
| 2 grade | 1.00 (reference) | |
| 3 | 3.26 | (0.42, 25.38) |
| 4 | 2.68 | (0.35, 20.24) |
| 5 | 5.33 | (0.72, 39.14) |
| 6 | 4.62 | (0.63, 33.87) |
| 7 | 4.85 | (0.66, 35.90) |
| 8 | 5.13 | (0.70, 37.68) |
| 9 | 3.93 | (0.52, 29.88) |
| Likelihood ratio statistics on 7 d.f. = 11.406, $P = 0.122$ | | |
| WHO grade | | |
| 1 | 1.00 (reference) | |
| 2 | 1.86 | (1.14, 3.04) |
| 3 | 2.20 | (1.34, 3.62) |
| 4 | 9.19 | (3.62, 23.32) |
| Likelihood ratio statistics on 3 d.f. = 20.113, $P = <0.001$ | | |
| Mild atypia | 1.00 (reference) | |
| Moderate atypia | 1.70 | (1.10, 2.62) |
| Severe atypia | 2.07 | (1.30, 3.30) |
| Likelihood ratio statistics on 2 d.f. = 10.063, $P = 0.007$ | | |
| AE1/AE3 | | |
| 0 | 1.00 (reference) | |
| 1 | 0.74 | (0.33, 1.67) |
| 2 | 1.04 | (0.48, 2.28) |
| 3 | 1.08 | (0.52, 2.25) |
| Likelihood ratio statistics on 3 d.f. = 2.701, $P = 0.440$ | | |
| NSE | | |
| 0 | 1.00 (reference) | |
| 1 | 0.67 | (0.39, 1.14) |
| 2 | 1.24 | (0.71, 2.16) |
| 3 | 21.66 | (2.53, 185.6) |
| Likelihood ratio statistics on 3 d.f. = 7.366, $P = 0.061$ | | |
| p53 | | |
| 0 | 1.00 (reference) | |
| 1 | 1.14 | (0.72, 1.81) |
| 2 | 1.17 | (0.43, 3.19) |
| 3 | 1.92 | (0.89, 4.15) |
| Likelihood ratio statistics on 3 d.f. = 2.550, $P = 0.466$ | | |
| PAP | | |
| 0 | 1.00 (reference) | |
| 1 | 0.96 | (0.13, 7.07) |
| 2 | 0.14 | (0.003, 0.72) |
| 3 | 0.12 | (0.003, 0.51) |
| Likelihood ratio statistics on 3 d.f. = 9.622, $P = 0.022$ | | |
| PSA | | |
| 0 | 1.00 (reference) | |
| 1 | 0.87 | (0.47, 1.61) |
| 2 | 0.90 | (0.50, 1.60) |
| 3 | 0.61 | (0.35, 1.05) |
| Likelihood ratio statistics on 3 d.f. = 6.682, $P = 0.083$ | | |

Table VII Multivariate stepwise Cox regression analysis stratified by M category (localised or metastatic stage) and resultant model. The 95% confidence limits are given in brackets

| Variable | Hazard ratio | |
|---|---|---|
| WHO | | |
| 1 | 1.00 (reference) | |
| 2 | 1.94 | (1.17, 3.19) |
| 3 | 2.13 | (1.29, 3.52) |
| 4 | 7.43 | (2.59, 21,31) |
| NSE | | |
| 0 | 1.00 (reference) | |
| 1 | 0.71 | (0.41, 1.22) |
| 2 | 1.07 | (0.61, 1.90) |
| 3 | 28.93 | (3.09, 271.3) |
| Age (years) | | |
| <59 | 1.00 (reference) | |
| 60-69 | 1.00 | (0.50, 2.04) |
| 70-79 | 1.28 | (0.65, 2.51) |
| >80- | 2.36 | (1.14, 4.87) |
| PAP | | |
| 0 | 1.00 (reference) | |
| 1 | 0.31 | (0.04, 2.72) |
| 2 | 0.17 | (0.03, 0.89) |
| 3 | 0.14 | (0.03, 0.59) |

1985), only glandular formation, nuclear anaplasia and the number of mitotic figures were significant parameters. Gallee et al. (1990) reviewed five different grading systems (Broders, Anderson, Gleason, Mostofi, Mostofi-Schroeder) and found that the best prognostic information, although not statistically significant, was obtained by the Mostofi-Schroeder and the Broders systems, while the Gleason system showed the least prognostic ability. Furthermore, Ten Kate et al. (1986) noticed that, compared with the complicated Gleason system, the less complicated grading systems resulted in the highest inter-observer reproducibility. Our data from the uni- and multivariate Cox regression model confirm that the WHO system (Mostofi system) is superior to the Gleason system in predicting survival, which is in agreement with Gallee et al. (1990) and Humphrey et al. (1991).

Like Schroeder et al. (1985), our univariate analysis demonstrates that cellular atypia predicts survival. Although we found significant correlations between the Gleason and the WHO systems and cellular atypia, the WHO system added prognostic information beyond that provided by the Gleason system and by cellular atypia in the stepwise Cox regression model. However, whereas most recent reports presenting good prognostication using the Gleason system have been performed on prostatectomy specimens, our series is based on pretreatment biopsies characterised by cauterisation artefacts (TUR-P specimens) and scanty material (core biopsies). As in our recent study of hormone-resistant prostatic carcinomas (Berner et al., 1993), we noticed some undergrading by the Gleason system in core biopsies.

Prostate acid phosphatase and PSA are produced both in benign and in malignant prostatic tissue and are widely used as tumour markers (Stamey et al., 1987). In contrast to benign epithelium, most reports on prostatic cancer tissue demonstrate heterogeneity in immunostaining of PAP and PSA and an apparent correlation between variation in stainability and tumour grade (Epstein & Eggleston, 1984; Sesterhenn et al., 1985; Feiner & Gonzales, 1986), which was also observed in our study. Like Sesterhenn et al. (1985) and Feiner and Gonzales (1986), we noticed that more specimens expressed reduced or no reactivity for PSA than for PAP. However, our uni- and multivariate analysis revealed PAP immunostaining to be a significant and independent factor in determining survival, which is in agreement with the findings of Sakai et al. (1991). PSA immunostaining, on the other hand, did not reach the level of statistical significance.

Approximately 50% of prostatic cancer specimens contain small numbers of neuroendocrine cells (di Sant'Agnese & de Mesy Jensen, 1987; Ro et al., 1987; Cohen et al., 1991), and it has been suggested that the demonstration of neuroendocrine features is an indicator of poor prognosis. Unlike di Sant'Agnese and de Mesy Jensen (1987) and Cohen et al. (1991), who used a series of different neuroendocrine markers, we applied only one monoclonal antibody raised against NSE, which may explain the lower scoring rate in our series (18%). Furthermore, neuroendocrine differentiation is mostly focal (Cohen et al., 1991) and small tissue specimens such as core biopsies may be inadequate in demonstrating such features. Thirty-eight per cent of the specimens in our study were core biopsies. Extensive NSE staining (+++) predicted poor outcome in the multivariate analysis. However, this was largely due to one core biopsy which histologically was a small-cell carcinoma. The 95% confidence interval was wide (Table VII), and the general significance of this result should therefore be viewed with caution. On the other hand, it has been reported (Ro et al., 1987) that small-cell carcinomas in the prostate frequently demonstrate neuroendocrine features. In a study of 20 small-cell carcinomas of the prostate, Têtu et al. (1987) reported a median survival of only 5 months. The patient in our study died 4 months after diagnosis of his prostate cancer.

The published data on any potential association between age and survival of prostate cancer are conflicting, and there is no general support in the literature for the concept that younger patients may have particularly aggressive tumours. Kant et al. (1992) reported a significant decrease in the relative 5 year survival in prostate cancer patients 75 years of age and older, and Partin et al. (1992) in a study of localised prostate cancer found age to be a significant and independent parameter by multivariate analysis. The findings of Kent et al. (1992) and Partin et al. (1992) of some relation between age and prognosis are in agreement with ours. However, this observation must be interpreted cautiously because of possible bias due to less intensive cancer treatment in elderly patients.

p53 mutations and p53 protein accumulation appear to be one of the most common features in several human neoplasms (Levine et al., 1991; Porter et al., 1992). In contrast to the short half-life, wild-type p53 protein present in normal tissues and undetectable immunohistochemically, the mutant form can be detected by immunohistochemical techniques. The biological significance of p53 overexpression is not yet established, but most authors agree that p53 protein expression occurs relatively late in neoplastic transformation. In prostate cancer nuclear p53 protein accumulation has been correlated with histological grade (Mellon et al., 1992), tumour progression (Berner et al., 1993) and DNA ploidy (Visakorpi et al., 1992). Van Veldhuizen et al. (1993) recently reported a predominant cytoplasmic staining pattern in 79% of a series of prostatic carcinomas. However, their negative controls were insufficient and their observations thus seem questionable. In our study only 17% of prostatic carcinomas demonstrated nuclear p53 protein accumulation. There was no correlation with survival or grade, although we found a weak trend in the univariate analysis indicating lower survival by increasing p53 protein accumulation.

Cytokeratins are a complex group of polypeptides that form cytoskeletal intermediate filaments specific for epithelial cells. Cytokeratin expression is found in both benign and malignant prostatic epithelium. The AE1/AE3 antibodies used in this study recognise high and low molecular weight cytokeratins (Woodcock-Mitchell et al., 1982). High molecular weight cytokeratins react in basal cells and may be a helpful criterion for the diagnosis of well-differentiated prostatic carcinomas (Sherwood et al., 1991). Like PAP and PSA, cytokeratins are markers for cell maturity. Like others (Feiner & Gonzales, 1986; Berner et al., 1993) we found reduced or absent expression of AE1/AE3 in poorly differentiated tumours.

Vimentin is a marker of mesenchymal tissues. However, coexpression of cytokeratin and vimentin has been found in normal prostatic epithelium (Leong et al., 1988; Nagle et al., 1991). Nagle et al. (1991) observed reduced vimentin staining in prostatic intraepithelial neoplasia and no vimentin reactivity in prostatic cancer tissue, whereas Leong *et al.* (1988) found positive vimentin staining in 83% of prostatic adenocarcinomas. In our study only 5% of the carcinomas were vimentin positive and the variable was not useful in the prediction of survival.

Although c-*erb*B-2 protein reactivity has been noticed in many different tumours, only a few studies have been performed on prostatic cancer tissue (McCann *et al.*, 1990; Ware *et al.*, 1991; Mellon *et al.*, 1992). Both Ware *et al.* (1991) and Mellon *et al.* (1992) used fresh material and found c-*erb*B-2 protein expression in 71% and in 21%, respectively, while McCann *et al.* (1990) did not observe c-*erb*B-2 protein expression in formalin-fixed tissue. Ware *et al.* (1991) also compared fresh and formalin-fixed tissue, and found that formalin fixation significantly reduced the c-*erb*B-2 protein immunoreactivity, which may explain the rather low c-*erb*B-2 protein reactivity (1.5%) in our series.

In summary, although modern immunohistochemistry certainly yields exciting insight into the development of prostate cancer, for the routine clinician the longer established variables such as patient's age, WHO grade, presence of distant metastases and PAP stainability give the most important prognostic information in the majority of patients. The fact that WHO grading was superior to Gleason grading and that the impact of PAP stainability exceeds that of PSA stainability needs further investigation.

This study was supported by The Norwegian Cancer Society.

### References

BAZINET, M., HARNDY, S.M., BEGIN, L.R., STEPHENSON, R.A. & FAIR, R.F. (1992). Prognostic significance of antigenic heterogeneity, Gleason grade, and ploidy of lymph node metastases in patients with prostate cancer. *The Prostate*, **20**, 311–326.

BERNER, A., NESLAND, J.M., WÆHRE, H., SILDE, J. & FOSSÅ, S.D. (1993). Hormone resistant prostatic carcinoma. An evaluation of prognostic factors in pre- and post-treatment specimens. *Br. J. Cancer*, **68**, 380–384.

BLUTE, M.L., NATIV, O., ZINCKE, H., FARROW, G.M., THERNEAU, T. & LIEBER, M.M. (1989). Pattern of failure after radical retropubic prostatectomy for clinically and pathologically localized adenocarcinoma of the prostate: influence of tumor deoxyribonucleic acid ploidy. *J. Urol.*, **142**, 1262–1265.

COHEN, R.J., GLEZERSON, G. & HAFFEJEE, Z. (1991). Neuroendocrine cells–a new prognostic parameter in prostate cancer. *Br. J. Urol.*, **68**, 258–262.

DI SANT'AGNESE, P.A. & DE MESY JENSEN, K. (1987). Neuroendocrine differentiation in prostate carcinoma. *Hum. Pathol.*, **18**, 849–856.

EPSTEIN, J.L. & EGGLESTON, J.C. (1984). Immunohistochemical localization of prostate-specific acid phosphatase and prostate-specific antigen in stage $A_2$ adenocarcinoma of the prostate. *Hum. Pathol.*, **15**, 853–859.

EPSTEIN, J.L., GERSON, P., EGGLESTON, J.C. & WALSH, P.C. (1986). Prognosis of untreated stage A1 prostatic carcinoma: A study of 94 cases with extended follow up. *J. Urol.*, **136**, 837–839.

EPSTEIN, J.L., PIZOV, G. & WALSH, P.C. (1993). Correlation of pathologic findings with progression after radical retropubic prostatectomy. *Cancer*, **71**, 3582–3593.

FEINER, H.D. & GONZALEZ, R. (1986). Carcinoma of the prostate with atypical immunohistological features. Clinical and histological correlates. *Am. J. Surg. Pathol.*, **10**, 765–770.

GALLEE, M.P.W., TEN KATE, F.J.W., MULDER, P.G.H., BLOM, J.H.M. & VAN DER HEUL, R.O. (1990). Histological grading of prostatic carcinoma in prostatectomy specimens. Comparison of prognostic accuracy of five grading systems. *Br. J. Urol.*, **65**, 368–375.

GLATTRE, E. & BLIX, E. (1980). *Evaluation of the Cause-of-Death Statistics*. Central Bureau of Statistics: Oslo.

GLEASON, D.F., MELLINGER, G.T. AND THE VETERANS ADMINISTRATION COOPERATIVE UROLOGICAL RESEARCH GROUP. (1974). Prediction of prognosis for prostatic adenocarcinoma by combined histological grading and clinical staging. *J. Urol.*, **111**, 58–64.

GULLICK, W.J., LOVE, S.B., WRIGHT, C., BARNES, D.M., GUSTERSON, B., HARRIS, A.L. & ALTMAN, D.G. (1991). c-*erb*B-2 protein overexpression in breast cancer is a risk factor in patients with involved and uninvolved lymph nodes. *Br. J. Cancer*, **63**, 434–438.

HALE, R.J., BUCKLEY, C.H., FOX, H. & WILLIAMS, J. (1992). Prognostic value of c-*erb*B-2 expression in uterine cervical carcinoma. *J. Clin. Pathol.*, **45**, 594–596.

HAMMOND, M.E., SAUSE, W.T., MARTZ, K.L., PILEPICH, M.V., ASBELL, S.D., RUBIN, P., MYERS, R.P. & FARROW, G.M. (1989). Correlation of prostate-specific acid phosphatase and prostate-specific antigen immunocytochemistry with survival in prostate carcinoma. *Cancer*, **63**, 461–466.

HERMANEK, P. & SOBIN, L.H. (1987). *UICC TNM Classification of Malignant Tumours*, pp. 141–144. Springer: New York.

HINTZE, J. (1992). *Manual of the Number Cruncher Statistical system Version 5.03*. Aysville, UT.

HSU, S.-M., RAINE, L. & FANGER, H. (1981). A comparative study of the peroxidase–antiperoxidase method and an avidin–biotin complex method for studying polypeptide hormones with radioimmunoassay antibodies. *Am. J. Clin. Pathol.*, **75**, 734–738.

HUMPHREY, P.A., WALTHER, P.J., CURRIN, S.M. & VOLLMER, R.T. (1991). Histologic grade, DNA ploidy, and intraglandular tumor extent as indicators of tumor progression of clinical stage B prostatic carcinomas. *Am. J. Surg. Pathol.*, **15**, 1165–1170.

JOHANSSON, J.-E., ADAMI, H.-O., ANDERSSON, S.-O., BERGSTRØM, R., KRUSEMO, U.B. & KRAAZ, W. (1989). Natural history of localized prostatic cancer. *Lancet*, **15**, 799–803.

KANT, A.K., GLOVER, C., HORM, J., SCHATZKIN, A., HARRIS, T.B. (1992). Does survival differ for older patients? *Cancer*, **70**, 2734–2740.

LEONG, A.S.-Y., GILGAM, P. & MILIOS, J. (1988). Cytokeratin and vimentin intermediate filament proteins in benign and neoplastic prostatic epithelium. *Histopathology*, **13**, 435–442.

LEVINE, A.J., MOMAND, J. & FINLAY, C.A. (1991). The p53 tumour suppressor gene. *Nature*, **351**, 453–456.

MCCANN, A., DERVAN, P.A., JOHNSTON, P.A., GULLICK, W.J. & CARNEY, D.N. (1990). c-erbB-2 oncoprotein expression in human primary tumors. *Cancer*, **65**, 88–92.

MELLON, K., THOMPSON, S., CHARLTON, R.G., MARSK, C., ROBINSON, M., LANE, D.P., HARRIS, C.H., HORNE, C.H.W. & NEAL, D.E. (1992). p53, c-erbB-2 and the epidermal growth factor receptor in the benign and malignant prostate. *J. Urol.*, **147**, 496–499.

MOSTOFI, F.K., SESTERHENN, I. & SOBIN, L.H. (1980). *Histological Typing of Prostate Tumours*. International histological classification of tumours. No. 22. World Health Organization: Geneva.

MURPHY, G.P., NATARAJAN, N., PONTES, J.E., SCHMITZ, R.L., SMART, C.R., SCHMIDT, J.D. & METTLIN, C. (1982). The national survey of prostate cancer in the United States by The American College of Surgeons. *J. Urol.*, **127**, 928–934.

NAGLE, R.B., BRAWER, M.K., KITTELSON, J. & CLARK, V. (1991). Phenotypic relationships of prostatic intraepithelial neoplasia to invasive prostatic carcinoma. *Am. J. Pathol.*, **138**, 119–128.

PARTIN, A.W., STEINBERG, G.D., PITCOCK, R.V., WU, L., PIANTADOSI, S., COFFEY, D.S. & EPSTEIN, J.L. (1992). Use of nuclear morphometry, Gleason histologic scoring, clinical stage, and age to predict disease-free survival among patients with prostate cancer. *Cancer*, **70**, 161–168.

PORTER, P.L., GOWN, A.M., KRAMP, S.G. & COLTRERA, M.D. (1992). Widespread p53 overexpression in human malignant tumors. *Am. J. Pathol.*, **140**, 145–153.

REILLY, S.M., BARNES, D.M., CAMPLEJOHN, R.S., BARTKOWA, J., GREGORY, W.M. & RICHARDS, M.A. (1991). The relationship between c-erbB-2 expression, S-phase fraction and prognosis in breast cancer. *Br. J. Cancer*, **63**, 444–446.

RO, J.Y., TÊTU, B., YALA, A.G. & ORDONEZ, N.G. (1987). Small cell carcinoma of the prostate II. Immunohistochemical and electron microscopic studies of 18 cases. *Cancer*, **59**, 977–982.

SAKAI, H., SHIRAISHI, K., MINAMI, Y., YUSHITA, Y. & KANETAKE, H. (1991). Immunohistochemical prostatic acid phosphatase level as a prognostic factor of prostatic carcinoma. *Prostate*, **19**, 265–272.

SCHROEDER, F.H., HOP, W.C., BLOM, J.H. & MOSTOFI, F.K. (1985). Grading of prostate carcinoma: multivariate analysis of prognostic parameters. *Prostate*, **7**, 13–20.

SESTERHENN, I.A., MOSTOFI, F.K. & DAVIS Jr, C.J. (1985). Immunopathology in prostate and bladder tumors. In: *Immunocytochemistry in Tumor Diagnosis*. Russo, J. (ed.) pp. 337–350. Martinus Nijhoff, Boston.

SHERWOOD, E.R., THEYER, G., STEINER, G., BERG, L.A., KOZLOWSKI, J.M. & LEE, C. (1991). Differential expression of specific cytokeratin polypeptides in the basal and luminal epithelia of the human prostate. *Prostate*, **18**, 303–314.

SMITH Jr, J.A., HERNANDEZ, A.D., WITTWEER, C.J., AVENT, J.M., GREENWOOD, J., HAMMOND, E.H. & MIDDLETON, R.G. (1991). Long-term follow-up after radical prostatectomi. *Urol. Clin. N. Am.*, **18**, 473–476.

STAMEY, T.A., YANG, N., HAY, A.R., MCNEAL, J.E., FREIHA, F.S. & REDWINE, E. (1987). Prostate-specific antigen as a serum marker for adenocarcinoma of the prostate. *N. Engl. J. Med.*, **317**, 911–916.

TEN KATE, F.J.W., GALLEE, M.P.W., SCHMITZ, P.I.M. & 4 others. (1986). Problems in grading of prostatic carcinoma: interobserver reproducibility of five different grading systems. *World J. Urol.*, **4**, 147–152.

TÊTU, B., RO, J.Y., AYALA, A.G., JOHNSON, D.E., LOGOTHETIS, C.J. & ORDONEZ, N.G. (1987). Small cell carcinoma of the prostate part I. A clinicopathologic study of 20 cases. *Cancer*, **59**, 1803–1809.

VAN VELDHUIZEN, P.J., SADASIVAN, R., GARCIA, F., AUSTENFELD, M.S. & STEPHENS, R.L. (1993). Mutant p53 expression in prostate cancer. *Prostate*, **22**, 23–30.

VISAKORPI, T., KALLIONIEMI, O.-P., HEIKKINEN, A., KOIVULA, T. & ISOLA, J. (1992). Small subgroup of aggressive, highly proliferative prostatic carcinomas defined by p53 accumulation. *J. Natl. Cancer Inst.*, **84**, 883–887.

WARE, J.L., MAYGARDEN, S.J., KOONTZ, W.W. & STROM, S.C. (1991). Immunohistochemical detection of c-erbB-2 protein in human benign and neoplastic prostate. *Hum. Pathol.*, **22**, 254–258.

WHITMORE Jr, W.F., WARNER Jr, J.A. & THOMPSON, I.M. (1991). Expectant management of localized prostatic cancer. *Cancer*, **67**, 1091–1096.

WOODCOCK-MITCHELL, J., EICHNER, R., NELSON, W.G. & SUN, T.T. (1982). Immunolocalization of keratin polypeptides in human epidermis using monoclonal antibodies. *J. Cell. Biol.*, **95**, 580–588.